# Supplementary material for: Are carbon emissions trading and green financial instruments synergistic? -Comprehensive quantitative research based on content analysis
Source: PLoS One. 2024 Mar 7;19(3):e0298601. doi: 10.1371/journal.pone.0298601 (PMC10919601; doi:10.1371/journal.pone.0298601)
Supplement: S1 Data — (ZIP) [file pone.0298601.s001.zip › Data/地级市Z_F工作报告.zip!/╔╜╬≈/╠1⁄2╘¡╩╨/└┤╫╘╣1⁄2╓┌║┼í╛┬φ┐╦╩2╛▌═°í┐_2006.docx]

各位代表：

我市已进入国民经济和社会发展第十一个五年规划时期，市政府根据市委八届十次全会通过的《关于制定太原市国民经济和社会发展第十一个五年规划的建议》，认真听取各方面意见，制定了《太原市国民经济和社会发展第十一个五年规划纲要（草案）》。现在，我代表市政府向大会作报告，请各位代表连同《纲要（草案）》一并审议，并请各位政协委员和其他列席人员提出意见。

一、“十五”时期国民经济和社会发展回顾

“十五”时期，全市人民在省委、省政府和市委的正确领导下，坚持以邓小平理论和“三个代表”重要思想为指导，全面落实科学发展观，紧紧抓住率先发展第一要务，开拓进取，扎实工作，全市经济社会持续快速协调发展，各项事业取得显著成就。

经济总量实现翻番，综合实力明显增强。全市地区生产总值连续跨越五个百亿台阶，达到895.5亿元，年均增长14%；财政总收入达到163亿元，年均增长23.7%；规模以上工业增加值达到292.1亿元，年均增长19.6%；固定资产投资达到438.5亿元，年均增长33.2%；社会消费品零售总额达到384亿元，年均增长15.2%；地区外贸进出口总额达到33.7亿美元，年均增长24.6%；城镇居民人均可支配收入达到10476元，年均增长11.7%；农民人均纯收入达到4402元，年均增长10.7%。主要经济指标超额完成了“十五”计划确定的目标。

结构调整顺利推进，工业经济快速增长。坚决淘汰落后产业，关停取缔土小企业和落后生产设施，大机焦比重达到87%，地方煤矿总数减少54%，煤炭回采率提高30个百分点。改造提升传统产业，实施重点调产和技改项目450项，太钢40万吨不锈钢、蓝星TDI一期、东方铝业8万吨电解铝等192个项目建成投产，太钢150万吨不锈钢、双喜轮胎180万套全钢子午胎、太锅集团450吨循环硫化床锅炉等重点项目进展顺利。规模以上工业利税增长2.6倍，利润增长8.7倍，工业万元产值能耗下降51.6%，经济效益综合指数由73.6提高到140.4。大力发展新兴产业，25项高新技术产业化项目列入国家示范工程项目，在省会城市中居第四位。高新技术产业增加值占工业增加值的比重达到20.7%。电子信息、新材料、食品医药等新兴产业正在成为新的支柱产业。培育壮大园区经济，不锈钢生态工业园、富士康工业园一期投用，后续建设进展顺利。高新区主要经济指标年均增速超过40%，发展速度在全国53个高新区中排第10位。经济区连续三年实现翻番，在全国54个国家级经济区中名列第一。民营区年均增速超过50%，非公有制经济占地区生产总值比重达到40.1%，6户民营企业进入全国民企500强。

　“三农”工作力度加大，农业基础得到加强。率先在全国省会城市中全部免征农业税。农村经济总收入达到410亿元，增长91.6%。农业产业化龙头企业发展到165家，以奶业、醋业、肉业、油脂业等为主的10条产业化链条基本形成。农产品加工产值达到40亿元，增长1.2倍，带动农户18万户，对农民人均纯收入的贡献率超过30%。乡镇企业增加值和上缴税金年均增长25.8%和43.7%。新增节水面积9.2万亩，治理水土流失75.5万亩。退耕还林106.8万亩，建设环城林带15.6万亩。新建改建县乡公路696公里，完成村村通油路2114公里，通油路、通班车行政村分别达到98%和95%。解决了41.5万人的饮水困难和水质不达标问题。转移农村富余劳动力12万人。8.8万农村贫困人口脱贫。两县四区开展农村新型合作医疗试点，参合率81.4%，覆盖人口35.4万。全面实行农村低保制度，2.5万农民享受低保政策待遇，覆盖面2.4%。

建设管理整体推进，城市环境逐步改善。累计投资150亿元，完成长风大街、黄河水源城市供水、汾河治理美化一期等34项城市基础设施和生态建设重点工程。完成50条城市主干道的建设改造、20条道路大修、150条小街小巷拓宽整治。新增集中供热面积2459万平方米，集中供热率73%。污水处理率63%，垃圾无害化处理率80%。旧城改造和经济适用房竣工258.4万平方米。国土收益34.6亿元，比“九五”增长6.5倍。持续开展城市环境综合整治，城市绿化覆盖率、绿地率分别达到36.1%和31.1%，人均公共绿地达到8.3平方米，被省政府命名为“省级园林城市”。汾河景区、城西水系分别荣获“迪拜国际改善人居环境最佳范例奖”、“中国人居环境范例奖”。坚持实施环保五大工程，空气质量二级以上天数由2000年的45天增加到2005年的245天，三项主要污染物指标年均下降16.4%。

改革开放不断深化，内外贸易繁荣活跃。完成市县两级政府机构改革。累计取消行政审批和收费项目600项，减幅达71%，审批环节和审批时间均减少65%。政务大厅审批事项窗口直接办结率30%，承诺件提前办结率66%，被评为“浙商最佳投资城市”之一，被中宣部列为全国信用建设宣传单位。深化国企改革，38户企业完成改制，93户转属改制，14户列入国家破产计划。长城微光、理工天成、晋西车轴成功上市。积极推进部门预算、国库集中收付、政府采购制度改革。粮食流通体制改革顺利推进。商贸流通蓬勃发展，形成了开化寺街、亲贤街等特色商业街区，涌现出一批大型超市和专业批发市场，各类商业网点发展到3.8万个。成功举办纪念太原建城2500年暨招商旅游年活动和三届国际面食节，新批外商投资企业151户，实际利用外资5.6亿美元，10家世界500强落户太原。对晋祠、天龙山、双塔景区进行综合整治，修复唱经楼、皇庙、拱极门。旅游收入年均增长29%。

群众利益有效保障，社会保持和谐稳定。城镇新增就业岗位36万个，19.1万下岗失业人员实现再就业，安置“4050”人员1.6万人，城镇登记失业率控制在4%以内。残疾人就业率88%。不断巩固“两个确保”，保障率和社会化发放率保持100%。养老保险、失业保险、医疗保险参保人数分别达到57.5万人、67.5万人和76.1万人。住房公积金制度覆盖79.1万人。城市低保标准由156元提高到200元，覆盖10.5万人。救济困难群众12.6万人次，发放救灾款3737.3万元。妥善安置拆迁居民2.3万户。廉租住房保障307户。清偿政府工程拖欠款9.5亿元，清偿率93.7%；清偿拖欠农民工工资1.18亿元，清偿率100%。城乡居民人均住宅建筑面积分别提高3.8平方米和2.6平方米。实行领导信访接待日制度，解决了一批重点信访案件。开展食品药品市场专项治理，整顿规范市场经济秩序。安全监管体系不断完善，安全生产形势保持平稳。加强社会治安综合治理，有力维护社会稳定。

各项事业全面进步，经济社会协调发展。实施科教兴市战略，财政科技投入年均增长42%，专利申请量年均增长19%，科技成果转化率由18%提高到25%，18项成果获国家奖项，制造业信息化、清洁能源两项试点通过国家验收。坚持教育优先发展，义务教育完成学制过渡，巩固了普九成果，全面普及学前三年教育，在全省率先基本普及高中阶段教育，高考升学率保持全省第一。建成市级以上示范高中16所，剥离企业学校36所，撤并边远山区中小学572所，改造农村中小学危房34.7万平方米。优质小学、初中分别达到36%和50%。对城乡贫困家庭子女实行“两免一补”。建成太原教育城域网和4所国家级重点职业学校、4个国家级技能型紧缺人才培养培训基地。启动教育园区建设。战胜了“非典”疫情。实施公共卫生项目12项。7项医学科研成果达到国际先进水平，28项国内领先。社区卫生服务网络覆盖率79%。人口自然增长率由9.7‰下降到4.27‰，被评为全省“人口和计划生育工作先进市”。成功举办三届晋阳文化艺术节和山西省第四届广场文化艺术节。承办了一批国际国内重大体育赛事，获得2008自行车BMX世锦赛暨奥运会预赛举办权。经济普查圆满完成。外事、气象、防震、档案、地方志、宗教、广电、新闻出版、国防后备力量建设等工作都取得新成绩，老龄、妇女、关心下一代等事业都有新进步。几年来，我市先后荣获“全国就业和再就业先进城市”、“全国双拥模范城”五连冠、“全国社区建设示范城市”、“全国优秀旅游城市”、“全国节约用水先进城市”、“全国人防先进城市”、“全国职业教育先进单位”、“全国实施体育锻炼标准先进城市”、“全国无偿献血先进城市”、“全国社会治安综合治理优秀城市”、“创建全国文明城市工作先进城市”和“全国民族团结进步模范集体”等称号。

民主法制不断加强，文明创建深入开展。各级政府主动向同级人大及其常委会报告工作，自觉接受人大监督。积极支持政协开展工作，加强同各民主党派、无党派人士和群众团体的联系，广泛听取各方面的意见和建议。省市人大代表建议和政协提案办结率100%。市政府提请市人大审议地方性法规35件，制订政府规章32件。积极开展法制宣传和法律援助，全民法制观念逐步增强。顺利完成第一届、第二届社区居委会换届和第六届、第七届村委会换届工作。深入开展精神文明创建活动，城乡文明程度不断提高。强化行政监察和审计监督，行风政风逐步好转，廉政建设和反腐败斗争取得积极成效。

各位代表，“十五”时期，是我市经济社会发展最快最好的时期之一，是城市面貌发生变化最大的时期之一，是城乡人民得到实惠最多的时期之一。这是在省委、省政府的正确领导下，市委、市政府带领全市人民团结奋斗的结果，是市人大、市政协和各位代表、各位委员监督支持的结果，是社会各界、国内外朋友积极参与开发建设的结果。我代表市政府向辛勤工作在各条战线的工人、农民、知识分子和各级干部，向大力支持政府工作的人大代表、政协委员、各民主党派、工商联、人民团体、无党派人士，向关心帮助太原发展的中央、省驻并单位和驻并部队、武警官兵，向积极参与太原建设的港澳台同胞、海外侨胞和国际友人，表示衷心的感谢！

在充分肯定成绩的同时，我们必须清醒地看到，我市经济社会发展中还存在不少问题和困难。主要是：干部群众的思想观念和行为方式与市场经济发展要求不能及时对接；改革落实不到位，影响经济社会发展的深层次体制性、机制性障碍还较多，对外开放严重不足；产业结构和增长方式还未根本性转变，技术创新体系不完善，自主创新能力需要大力增强；城乡发展不够协调，农村发展和农民增收的长效机制还未形成；基础设施建设跟不上城市的快速发展，绿化美化净化水平不高，城市面貌和形象与省会城市地位不相适应；结构性污染突出，生态环境比较脆弱，保护和提升人民生存质量的压力较大；社会事业发展滞后，公共产品和公共服务还不能满足群众日益增长的需求；一些部门和工作人员的服务水平、办事效率与群众的要求还有差距，政府职能转变需要进一步加快。对于这些问题和薄弱环节，我们要认真分析，科学应对，重力点击，采取扎实有效措施，不断加以解决，以不辜负人民群众对政府的信任和厚望！

五年的丰富实践，使我们深刻体会到：要把太原的各项工作做好，必须牢牢抓住率先发展第一要务，全面落实科学发展观，努力实现既快又好的发展；必须深入推进产业结构调整，转变经济增长方式，发挥比较优势，走新型工业化道路；必须坚持改革开放，用改革的办法解决前进中遇到的问题，为各项事业注入新的生机与活力；必须把广大人民群众的利益放在首位，充分调动人民群众的积极性和创造性，努力构建和谐社会；必须坚持依法行政、科学行政、民主行政，转变政府职能，提高办事效率，为经济社会发展营造良好环境。

二、“十一五”时期的指导思想和奋斗目标

　“十一五”时期，是我市全面建设小康社会的重要时期，也是建设创新型城市、实现率先发展战略目标的关键时期。当前，我们面临很好的发展机遇：经济全球化和区域经济一体化进程进一步加快，生产要素和产业加速流动，有利于我们依托资源优势，承接国外和沿海地区产业转移，提升竞争力；国家实施中部崛起和振兴老工业基地战略，有望把我市列入享受东北老工业基地有关政策的城市，有利于我们获得更多的政策支持；省委省政府进一步支持太原在全省率先发展，做出建设太原经济圈和大运经济带的战略决策，有利于我们增强发展动力，拓展发展空间；通过几年结构调整，我市经济增长方式不断转变，自主发展能力进一步增强，为“十一五”发展打下了坚实的基础。未来几年，我市将继续处于平稳快速的发展区间。同时，我们也面临发展压力增大、不确定因素增多、各项任务繁重的严峻挑战。我们必须振作精神迎难而上，昂扬奋进追求卓越，紧紧抓住有利机遇，坚持科学发展，突出创新发展，实现率先发展，巩固和保持“十五”以来快速健康发展的良好势头，推动经济社会发展再上新台阶。

“十一五”时期我市经济社会发展的指导思想是：高举邓小平理论和“三个代表”重要思想伟大旗帜，全面落实科学发展观，坚持用建设创新型城市总揽率先发展的各项工作，以“五个创新”为突破，以绿色转型为路径，以改革开放为动力，以科教兴市、人才强市为支撑，大力调整经济结构，努力转变增长方式，着力统筹城乡发展，致力构建和谐社会，把太原建成经济发达、文化繁荣、生态环境良好、社会文明和谐、人民生活殷实的新型工业基地、文化名城和现代化都市，跨入全国先进省会城市行列。

根据这一指导思想，今后五年，经济社会发展要遵循以下原则：

——坚持以科学发展观指导下的率先发展为主题。紧紧扭住发展第一要务，千方百计加快发展。切实转变发展观念，创新发展思路，丰富发展内涵，完善发展举措，提升发展水平，更加注重经济运行的质量和效益，努力实现经济社会又快又好发展。

——坚持以推进结构调整、转变经济增长方式为主线。整体推进绿色转型，走新型工业化道路，促进产业结构优化升级，使发展建立在提高质量效益、高效利用资源、减少环境污染的基础上，使经济增长与结构、质量、效益相统一，经济发展与人口、资源、环境、生态相协调，走绿色文明发展之路。

——坚持以改革开放和全面创新为动力。坚定不移地深化改革，扩大开放，为经济社会发展注入活力。坚定不移地实施科教兴市和人才强市战略，完善区域科技创新体系，增强自主创新能力；坚定不移地推进思想观念创新、体制机制创新、发展环境创新、文化氛围创新，为率先发展提供不竭动力。

——坚持以城市与农村、经济与社会协调发展为重点。更加重视解决“三农”问题，统筹配置城乡资源，以城带乡、以工促农，大力推进新农村建设，逐步缩小城乡差距。加快发展社会事业，加大对公共事业、公共服务和社会管理方面的投入，创造有利于人的全面发展的良好环境，实现城市与农村、经济与社会互为促进，协调发展。

——坚持以不断提高人民生活水平为目的。把维护和实现广大人民的利益作为一切工作的出发点和落脚点，注重社会公平，努力扩大就业，完善社会保障体系，提高人民生活质量，让改革发展的成果惠及全体人民。

按照上述指导思想和基本原则，根据省“十一五”规划指标体系，我市“十一五”期间将建立“绿色高压线”，用绿色经济发展指标体系和绿色指标考核体系指导、推动经济社会发展，考核评价各县区、各部门的发展水平和工作业绩。新的指标体系力求体现科学发展观和构建和谐社会的基本要求，符合太原经济社会发展实际，具有较强的导向性和可操作性。这一指标体系，由五个方面四十四项指标组成。

——经济增长方面，地区生产总值年均增长12%；人均地区生产总值年均增长10.5%；全社会固定资产投资年均增长14%；外来直接投资年均增长18%，其中境外直接投资年均增长20%；财政总收入年均增长15% ，一般预算收入年均增长13%；粮食总产量年均25万吨；第三产业增加值占地区生产总值比重达到52%。规模以上工业增加值年均增长13%；社会消费品零售总额年均增长15%；居民消费价格总水平上涨幅度控制在3%以内；外贸进出口总额年均增长15%。

——社会发展方面，到2010年，人口自然增长率控制在4.5‰以内；高中阶段毛入学率90%，高等教育毛入学率40%；县乡村三级医疗卫生机构达标率80%以上；每千人拥有医生4.5人、病床5.5张；新型农村合作医疗覆盖率 100%；每万人公共馆藏图书5万册；人均公共体育场馆面积1.2平方米；城镇化率86%；城镇登记失业率控制在4%以内；城镇基本社会保障覆盖率82%；每百人拥有电话（固定、移动）150部；每千人拥有计算机273台；亿元地区生产总值生产安全事故死亡率年均下降8.3%，五年下降35%；地方煤炭生产百万吨死亡人数下降到0.8人，年均下降16.1%；公路密度达到53.3公里/百平方公里；各级财政对科技、教育、农业投入的增长幅度高于财政经常性收入增长幅度。

——科技进步方面，到2010年，研究与开发经费占地区生产总值比重达到2%；高新技术产业增加值占地区生产总值比重达到12%；每10万人专利申请数达到60项；新型工业化水平达到50%以上。

——资源环境方面，森林覆盖率达到17%，年均提高1个百分点；城市建成区绿化覆盖率达到41%，年均提高1个百分点；城市生活垃圾无害化处理率达到90 %，污水处理率达到90%；城市集中供热普及率达到90%；万元地区生产总值污染物排放总量年均下降 15.8%，五年下降57.6%；万元地区生产总值综合能耗年均下降6.9%，五年降低30%；万元地区生产总值平均耗水量年均下降3%，五年下降14.3%；矿区煤炭资源回采率达到60%；耕地保有量166万亩左右。

——人民生活方面，城镇居民人均可支配收入年均增长10%；农民人均纯收入年均增长11%；占人口20%的低收入者收入增长率城镇年均增长11%以上，农村年均增长12%以上；到2010年，城镇居民人均住宅建筑面积达到27平方米，农村居民人均住宅建筑面积达到32平方米；人口预期寿命达到76岁。

各位代表，我市“十一五”规划的发展目标既考虑需要和可能，又兼顾当前和长远，科学严谨，切实可行。只要我们坚定信心，同心同德，奋发进取，这一宏伟蓝图一定能够如期实现！

三、深化经济结构调整，构建省会城市特色产业体系

把结构调整作为落实科学发展观的切入点，强化绿色理念在结构调整中的指导作用，促进经济结构绿色转型，努力构筑以绿色产业为主体，新兴产业、高科技产业为主导，先进制造业为支撑，服务业全面发展的具有国际竞争力的省会特色产业体系。

发挥比较优势，培育壮大工业支柱产业。按照大项目、产业链、产业群和产业基地发展方向，优化产业布局，把优质冶金、装备制造、清洁能源、精细化工、电子信息、新材料、现代医药等产业培育成代表太原工业水平、竞争力较强的支柱产业。冶金工业，严格控制淘汰初级产品，加快发展不锈钢、冷轧硅钢、汽车板、铝镁合金、优质铝板带为主导的系列产品，重点支持太钢150万吨不锈钢、新东方铝业二期。建成全球最大和最具竞争力的不锈钢生产基地，火车车轴钢、军工钢、纯铁等重点品种市场占有率居全国第一。机械装备制造业，重点突破载重汽车整车和矿山机械成套设备两大难点，巩固提升重型机械、铁路机械、基础机械三大领域优势产品，整合培育精密锻铸件、铝镁合金压铸件、磁性材料及深加工三大产业集群。扶持重型汽车、太重智能化起重机、晋机70吨重载敞车、铁路车辆用车轴及轮对技术改造等重点项目，建成国家新型装备制造工业基地。清洁能源工业，按照“关小建大、资源整合、提升改造”的思路，淘汰30万吨以下煤矿和非综采的煤矿，煤矿数量减少到100个左右，支持大型煤矿企业兼并、收购现有小煤矿资源，通过整合进行产能置换，发展一批现代化大型矿井和大型企业。不再新批生产商品煤的煤矿，只给新上煤电一体、煤化工、煤制油等项目按市场机制配置适当的资源。严格控制焦炭总量，焦化企业数量缩减到21户，减少40%，焦炭产量调控在1500万吨左右，大机焦比例占到90%以上。支持城市集中供热、矸石电厂等项目建设。重点支持古交电厂二期、二电厂六期和港源、万光等余热利用发电机组项目。建设和完善城市电网。化工工业，以煤化深加工为突破口，大力发展煤的气化、液化、焦化和煤焦油化工、电石乙炔化工、碳化工及煤炭的清洁利用。依托清徐、古交，规划建设一批焦炉煤气转化化肥、甲醇及下游产品、煤焦油加工方面的大项目、大企业。重点支持双喜轮胎180万套全钢子午胎、侨友化工8万吨粗苯萃取精制和1万吨富马酸、三兴焦炉煤气净化、金业、古交一一粗苯回收提升改造项目以及甲醇、合成氨、煤焦油深加工等项目，精细化工率达到60%。电子信息产业，大力提升电子元器件生产成套设备集成能力，重点发展软件和系统集成产品。扶持智能自动控制设备、新型电子元器件集成设备、数字音视频设备产业化和光电深加工产品开发等重点项目。新材料产业，加快发展高性能陶瓷材料、高技术复合耐火材料和纳米碳酸钙为主的新材料工业。对钕铁硼磁性材料关键生产技术进行攻关，大力开发高端产品，建立钕铁硼磁体——磁体精加工——信息永磁产品和永磁电机产业链。扶持镁铝合金加工业的规模化发展，提高深加工能力，努力建设世界镁合金加工中心。现代医药业，重点发展化学药品、中成药、生物制药和生化药品，形成一批具有自主品牌和地方特色的名优特新产品。到2010年，七大产业工业总产值达到1870亿元以上，比“十五”期末翻一番。

大力发展第三产业，优化三次产业结构。围绕建成全国商贸物流中心城市、全省旅游中心城市目标，大力发展现代商贸流通业、现代服务业和文化旅游业，提高第三产业占国民经济的比重。现代商贸流通业，要完善城市商业网点功能，改造提升传统商贸流通业。加快发展连锁经营、物流配送和电子商务等现代流通方式，鼓励有条件的连锁企业通过兼并联合、资产重组、特许经营等方式扩大经营规模，支持大型商贸企业向购物中心和综合超市发展。抓好武宿综合物流园区、万柏林综合物流园区、唐久超市物流园区、再生资源回收利用市场等重点工程，构建立体、快速、安全的现代物流体系。加快农村商业网点建设，实施“万村千乡”工程，建设一批大型农产品批发市场和标准化农产品超市，推动城市商贸流通网络向农村延伸，促进城乡物流交换。现代服务业，要加快构筑与经济社会发展相协调的现代服务体系，重点发展金融保险、邮电通讯、信息咨询等现代服务业，促进服务业的规模扩张与质量提升。积极发展家政服务、托老托幼、社区保安、保洁保绿等新兴服务业，拓宽社区服务领域，满足群众的多层次需求。大力发展会展经济，通过引进、培育、合资、合作等方式，整合会议、场馆等展览业资源，积极举办全国性、行业性会展和文化、体育、经贸等大型活动，培育有特色、有影响的会展品牌，办好国际面食节等节会活动。文化旅游业，要加快完善旅游产业体系，重点建设晋祠、崇善寺、迎泽湖、文瀛湖等精品景区，抓好双塔寺、天龙山文化旅游区、九龙山旅游度假区、云顶山森林公园等旅游景区的建设管理，重点开发历史文化游、商务会展游、休闲度假游和产业观光游，大力开发文化娱乐旅游，挖掘优秀文化资源，丰富旅游项目和内容，满足游客多方面的需求。围绕游、购、娱、吃、住、行，统筹整合旅行社、酒店、餐饮、交通、文化娱乐等行业的发展，建设一批能与国际接轨的旅行社、五星、四星级酒店。充分发挥旅游中心城市的集散功能和辐射带动作用，加强与周边城市的合作，形成借势发展、合作共荣的良性发展格局。搞好旅游促销和市场开发，扶持大型旅游企业。到2010年，旅游总收入达到163亿元，占生产总值的比重达到10%左右。实施文化强市战略，积极申报国家历史文化名城，推出具有晋阳文化特色的先进文化产品。深化文化体制改革，整合资源，优化结构，加快新闻出版、广播影视、文艺演出等领域经营性事业单位的体制转换，做强做大文化广播集团和报业集团，推进文化产业跨越式发展。

壮大园区经济，促进产业集聚发展。坚持把园区发展作为结构调整的重要载体，建设设施配套、功能健全、环境优良、资源共享、分工协作的工业园区。按照“同业入园、形成循环”的原则，建设专业生态园，实现生产要素的集约经营，形成产业集群优势。高新区要高起点规划，拓展发展空间，围绕建设国家级电子信息产业基地、高科技产品出口基地和煤化工技术研发基地的目标，突出抓好电子装备与信息产业、新材料与新能源产业、环保与高效节能产业三大产业95个项目。把留学人员创业园、火炬创新创业园和软件园建成国家级品牌园。高新技术企业总量占到全省的65%，主要经济指标每年增长20%以上，成为新型产业示范区和自主创新示范区。经济区要围绕建设新兴产业规模化示范区的目标，以IT、新材料加工、装备制造、生物制药、食品及农产品加工、新型建材等六大产业为基础，抓好配套建设和跟踪服务，积极发展拥有完整产业链条和核心技术的产业群，主要经济指标每年增长60%以上，进入中西部一流国家级经济区行列。民营区要依托大运经济带，加强产业规划和资源整合，加快南区建设，大力发展服装、物流等都市型、生态型产业。搞好阳曲新区规划和建设，培育一批销售收入上亿元的大型民营企业，把民营区建成以新材料、机械制造、食品医药为主体的新兴工业产业基地，主要经济指标每年增长40%以上。富士康科技工业园要加快建设进度，构筑产业集群，壮大经济规模，突出在IT产业中的龙头作用，建成先进制造业示范园，到2010年实现产值300亿元。不锈钢生态工业园要聚集优强企业，建成集不锈钢产品生产、加工、集散为一体的精品工业园，到2010年不锈钢转化能力达到50万吨，实现销售收入150亿元。加快太原铝镁工业加工区、太原服装加工园、万柏林装备机械加工园、太原信息产业园等专业园区的基础设施建设，提高入园企业数量和质量，尽快形成集聚效应。

优化企业组织结构，形成梯次发展的优势企业群。以重大项目投资和合作为依托，通过技改、联合、嫁接、重组、上市等途径，推进企业资金、技术和人力资源整合，重点培育和发展一批技术进步、主业集中、比较优势和规模优势突出的大型企业集团。把太钢、西山、太重、晋机、富士康、太化等企业培育成销售收入超过100亿元的国家级龙头企业；把煤气化、美锦、双喜轮胎、新东方铝业、恒通能源等企业培育成销售收入50—100亿元的省级龙头企业；把卷烟、汾机、机车、刚玉、锅炉、狮头、金业、梗阳、垣跃、理工天成等一批企业培育成销售收入10—50亿元的市级龙头企业，形成梯次发展的优势企业群。实施名牌发展战略，打造一批具有核心竞争力的名牌产品。

四、统筹城乡协调发展，大力推进社会主义新农村建设

把推进新农村建设作为全面建设小康社会的突破口，工作重点和财政投入向农村倾斜，坚持“多予、少取、放活”的方针，加快建立工业反哺农业、城市支持农村的体制机制和政策措施，开辟农业增效、农民增收的有效途径，不断改善农村生产生活条件和整体面貌，努力形成城乡一体、共同繁荣的格局。

整治村容村貌，完善基础设施。按照“生产发展、生活宽裕、乡风文明、村容整洁、管理民主”的要求，认真组织实施“百村试点、千村美化”工程，建设以沼气为主的康庄生态家园。全市新农村建设要以“四化、四改”为重点，改善农村基础设施和农民居住环境。“四化”是：街巷硬化，解决农民群众行路难的问题；村庄绿化，营造良好的生活环境；环境净化，重点整治脏、乱、差；路灯亮化，主要街道安装路灯、配套照明设施。“四改”是：改水，使农民逐步饮用上卫生安全的自来水，农村饮水安全人口达标率达到90%；改厨，积极发展沼气等清洁能源，用燃气灶逐步取代现行的烧煤、燃柴灶具；改圈，做到人畜分离，提倡发展养殖小区；改厕，取消露天粪坑，逐步提高卫生厕所的比例。加快农村路网改造，新建改造县乡村公路2000公里，三县一市建设二级客运站，所有行政村通油路、通班车。完成村村通电话、通广播电视，发展农村互联网用户。科学制定小城镇发展规划，准确把握功能定位，完善城镇职能，培育产业集群，把黄寨、清源、徐沟、晋祠、马兰、北格等中心镇建成优势明显、各具特色、辐射强劲的小城镇。让广大农民在新农村建设中见到成效、得到实惠。

立足产业支撑，广辟增收渠道。以增加农民收入为核心，大力实施龙头企业特色县的县域经济发展战略，按照特色立县、项目强县、放权活县的思路，支持各县区发挥比较优势，把主导产业发展、龙头企业培育和县乡财源建设结合起来，提高县域经济的规模、质量和效益。大力发展现代农业，加快农业结构调整，重点抓好优质粮、食用菌、无公害蔬菜、畜牧养殖、苗木花卉、中药材、瓜果7大生产基地建设。“十一五”期末，农村经济总收入达到600亿元，年均增长9%。积极推进农业产业化经营，抓好醋业、奶业和肉制品、果菜、粮油为主的加工业，扶持水塔醋业、旭光薯业、森泰牧业等一批绿色农业龙头企业做大做强。建设农产品加工园区，抓好蒙牛入并项目，整合省城农业资源，鼓励城市资本向农村流动，提高农产品竞争能力。到2010年，农产品加工产值达到100亿元，成为全省农产品加工中心城市。大力发展各级各类农村经济合作组织和中介组织，完成河西农副产品市场改扩建工程，完善农产品市场体系。支持乡镇企业创新发展，增加值和上缴税金年均增长18%以上。积极发展农村二、三产业，提高农民非农收入比重。引导8万农村富余劳动力向非农产业和城镇转移就业，从事第一产业的劳动力下降15%。加大扶贫开发力度，以移民并村为重点，突出抓好汾河库区整体搬迁和娄烦、阳曲15个村整村推进。今年帮助农村2.7万贫困人口实现脱贫，移民搬迁9276人。积极争取国家对老区的支持政策，实施“关爱老区、帮扶百村”扶贫建设工程，人均收入千元以下的老区村，农民人均纯收入年均增长20%以上，娄烦在全省率先实现脱贫。大力改善农业生产条件，实施汾河流域高效农业示范区建设，改造中低产田40万亩。积极推进精品小流域建设，治理水土流失100万亩。建设高标准农业节水园区20个，新增节水面积10万亩。大力推进农业机械化，机械化综合作业水平达到50%以上。

建立保障机制，促进全面进步。坚持以城带乡、以工补农、政策扶农，形成长效机制，确保农村社会事业全面进步。完善农村土地制度，稳步推进土地使用权流转，依法保障农民承包土地的各项权利，发展适度规模经营。改革征地制度，建立保护农民土地权益的长效机制。巩固农村税费改革成果，按照“减轻、规范、稳定”的要求，全面执行各项政策措施。加大配套改革力度，推进乡镇机构和县乡财政管理体制改革。健全农业社会化服务体系，改善农村富余劳动力转移就业环境。创新农业科技服务体系，实施农业科技入户工程和科普惠农工程，为农民提供先进适用技术，实用新技术覆盖率达到95%，科技对农业的贡献率超过50%。整合农村教育资源，重视发展农村学前教育，稳定农村幼教队伍，建设高标准九年一贯制学校和寄宿制中心小学，大力发展农村中小学远程教育，农村标准化学校达到80%以上，十县区全部通过省义务教育标准化验收。大力发展农村职业教育，完成劳动力转移引导性培训25万人，职业技能示范性培训10万人，培养一批有文化、懂技术、会经营的新型农民。加大卫生支农力度，更新乡镇卫生院医疗设备，每个行政村都设置卫生所，方便农民看病治疗。开展“亿万农民健康促进行动”，实行对口支援和巡回医疗制度，扩大农村医疗救助范围，落实乡村卫生人员待遇和农村计划生育家庭奖励政策。以被征地农民、进城务工农民、乡镇企业职工为重点，扩大农村养老保险覆盖面，让更多的农民享受现代城市文明成果。

五、强化城市规划建设管理，提升城市承载能力

坚持以人为本的城市发展理念，构建人与自然和谐相长的绿色城市环境。突出“汾河景观、文化品位、立体交通、新区引领”四大亮点，不断完善城市功能，提升城市品位，把太原建设成为更适宜人居、更适合发展、更适于创业的生态宜居城市。

科学规划，体现新理念。强化规划的龙头作用，处理好近期与长远、局部与整体、经济建设与社会发展、城市建设与环境保护、现代化建设与历史文化遗产保护等关系，高标准完成2006—2020城市总体规划修编和2006—2010近期建设规划编制工作，做好社会公共事业规划、城市基础设施专项规划和重点地区控制性详细规划。加大规划监督力度，对规划区范围内的建设用地和建设活动实行统一、严格的规划监督管理，切实增强执行规划的刚性约束，使城乡的开发建设置于规划的严格控制之下，维护规划的严肃性和权威性。严格规划责任追究制度，坚决查处违反规划的行为，确保规划的顺利实施。

扩容提质，打造新亮点。按照“南移西进、北展东扩”的思路，疏解旧城，建设新区。着眼于提高城市形象和品位，启动以山西国际会展中心、市政中心为主的长风商务区建设，高标准规划、建设汾河两岸建筑和景观，打造代表太原新形象的亮点片区。以汾河和迎泽、长风大街为主轴，以历史遗存为重点，挖掘城市文化内涵，形成一批文化景观群。建设太原音乐厅、太原博物馆、太原档案馆、太原广播电视中心等大型标志性工程，形成一批精品建筑群。着眼于增强城市承载能力，新建太原—古交、古交—娄烦—吕梁界高速公路，建设西山运煤专线，推进石太客运专线、太中银铁路、太古岚铁路建设，支持4E级国际机场改造工程，做好建设贯穿城市南北并接轨榆次的“三纵三横”轻轨的前期研究和准备工作。着眼于改善城市生态环境，实施汾河治理美化二期、城西水系二期、晋阳湖改造开发等重点工程。积极开展城市电气化建设，逐步改变能源消费结构，努力把太原建成全国第一个电气化城市。着眼于建设现代化商业都市，提升传统特色商业街区的档次，加快长风街城南商业中心建设，形成购物、餐饮、休闲消费的品牌街区。

提升基础，构筑新框架。构筑一流省会城市现代化基础设施框架，加快长风大街东延、滨河东西路南北延、体育路南延进度，实施中环立体快速路、长风大街西延、机场大道、中环桥、学府桥等重点工程，对大同路、南内环东街、胜利街、双塔街等主干道进行拓宽改造，城市道路总里程达到1827公里。改造供水管网，日供水能力达到152万立方米。增强污水处理能力，建设城南污水处理厂，实施河西北中部污水处理厂二期、北郊污水处理厂二期及回用工程，完成城市“六河”综合治理美化和太钢、南城地区防洪工程，更新改造城市雨污水管网，实现城市道路、河渠雨污分流排放。完善垃圾分类收运、处理系统，完成垃圾焚烧发电工程，建设河西垃圾中转站、侯村垃圾卫生填埋场，逐步实现垃圾无害化、减量化和资源化。加快热源厂建设，大力推进集中供热，年均扩网1000万平方米以上。推进天然气开发利用，城市气化率达到98%以上。建设客运北站和西站。对小街小巷和旧住宅区进行改造和整治，打通断头路，畅通微循环。加快无障碍设施建设。加大“城中村”改造力度，2010年中心城范围内的城中村基本改造完毕。旧城改造80万平方米，经济适用房完成200万平方米。抓好煤矿沉陷区、矿工棚户区的治理改造，三年新建住宅150万平方米，让2.3万户、7.1万人告别沉陷区和棚户区，住进新区、新村、新楼房！

强化管理，展现新容颜。完善城市管理体制，按照责权统一、重心下移、立足基层、属地管理的思路，明确城区在城市管理中的主体地位，强化街道、社区在城市管理中的基础作用。调整综合执法的区域关系和职能权限，实现全方位、多层次的监控管理，提高执法效率。对市民关心的热点、难点问题和重大城市管理决策，通过公开听证、意见征询、热线电话等途径，主动接受市民监督。落实绿化管养、环境卫生责任，建立健全分级巡查、责任监督、绩效考核等制度。深入推进城市环境综合整治，大力开展爱国卫生运动，全面推行小区物业化管理，营造干净整洁的卫生环境，创建全国卫生城市。我们坚信，未来五年，太原的容颜会更加青春、更加靓丽，一座功能完备、环境优雅、繁荣发达、文明进步的现代化园林城市必将矗立在世人面前！

六、转变经济增长方式，建设创新型城市和资源节约型、环境友好型城市

在科学发展观指导下，摒弃传统的发展理念，大力发展绿色经济，倡导绿色生产方式、生活方式，追求经济效益、社会效益和生态效益共同可持续增长的发展模式，坚决不以浪费资源、破坏环境为代价发展经济。

建立健全创新体系，切实增强自主创新能力。把提高自主创新能力作为转变增长方式和建设创新型城市的中心环节，深化科技体制改革，形成与市场经济体制相适应的科技管理体制和创新机制。建立健全以企业为主体、市场为导向、产学研相结合的技术创新体系。针对经济社会发展中的技术瓶颈，加快不锈钢深加工、机械装备制造、精细化工和能源环保等传统产业共性技术、关键技术的集成创新，强化新材料、电子信息、食品医药等高新技术产业核心技术的研发，突出节能降耗、污染防治、生态治理等领域的技术攻关，形成一批具有自主知识产权的科技成果。推进跨地区、跨行业的技术创新合作，引进一批先进适用技术，提高消化吸收再创新能力。政府要建立企业技术创新扶持基金，鼓励和扶持大中型企业建立技术中心，积极组建行业工程技术中心，不断增强企业自主创新能力。大力推进民营企业科技进步与创新，扶持发展一批科技型民营企业。再创建国家级企业技术中心1个、省级企业技术中心6个，认定市级企业技术中心40个以上。重点建设技术研发公共服务平台、成果转化服务体系和“一网两库”服务平台。强化知识产权保护工作，夯实科技创新基础，专利授权量年均增长30%，科技成果转化率达到40%以上。加强对技术创新人才的发现、培育、爱护和引进工作，在全社会营造尊重知识、尊重人才、尊重创造的良好氛围。

积极发展循环经济，大力建设生态工业园区。制定发展循环经济、推行清洁生产规划，加快推进试点工作。对能耗、物耗、水耗高和污染排放严重的重点企业强制实施技术改造，对年排水量30万吨以上企业实施废水“零排放”工程，对高污染行业实施全行业清洁生产强制审核。大力提升煤炭、焦化、冶金、电力、建材5大产业资源能源利用水平，煤矸石综合利用率提高10个百分点，电厂粉煤灰利用率提高15个百分点，焦油、粗苯回收率达到100%，焦炉剩余煤气回收综合利用率达到100%，焦化废水全部实现闭路循环，工业用水重复利用率达到96%。把高新区、民营区、不锈钢工业园区、信息产业园建成生态示范园区，经济区建成省级循环经济示范区，所有园区都要按照循环经济模式进行规划和布局。

全方位推行节能措施，加快节约型社会建设步伐。认真落实国家节能中长期规划，做好节能、节水、节材、节地等专项规划，努力形成节约型生产模式、消费模式和城市建设模式。制定科学的耗能指标，加强对年耗能万吨标准煤以上企业的监督管理，大力推广工业节能降耗先进技术和设备。搞好交通运输和农业机械节能，逐步使用清洁能源车辆。全面推进建筑节能，到2010年全市新建建筑达到节能65%的标准。新建民用建筑供热、供水实行“分户控制、分户计量”，逐年改造既有建筑。加强水资源保护，继续关停自备井。开展企业节水技术改造，推广节水设备和器具。搞好再生水利用，建设一批中水回用工程和城市雨水积蓄利用示范工程，构建节水型城市。加强原材料消耗管理，提高材料强度和使用寿命，严禁过度包装。推进木材节约和代用，扩大不锈钢材料应用范围。实行最严格的土地保护制度，推进建设用地节约集约利用，新型墙体材料比例达到80%以上。

严把环保门槛，确保社会生态安全。要以壮士断腕的勇气，加大对生产领域环境污染的治理力度，把环境成本纳入企业生产经营成本，使企业切实负担起维护生态安全的责任。坚决取缔浪费资源、污染环境的工艺、设备和企业，坚决淘汰高投入、高消耗、高污染项目，把好环保准入关。完成电厂烟气脱硫等一批重点治理项目，严格控制污染物排放总量，工业污染源实现全面稳定达标排放，工业固体废物处置利用率和废水排放达标率达到95%。开展生态功能区划工作，合理调整工业布局，逐步搬迁市区企业。大力调整燃料结构，推广使用清洁能源，不断扩大无燃煤区范围。规范各种作业行为，对固体废弃物、城乡结合部、煤焦发运站、建筑工地、清扫保洁、车辆运输抛洒进行不间断专项整顿，加大扬尘污染和机动车尾气污染防治力度，严格执法监督，严厉查处环保违法行为。三项污染物平均下降31%，二级以上天数突破300天，努力创建全国环保模范城。

高质量推进城乡绿化，着力建设北方绿洲。以植树造林和园林绿化为重点，建设点线面有机结合、功能良好的城乡生态系统。严格按照绿地系统规划进行建设项目审批，在旧城区开展园林化单位、社区创建活动，栽植添绿，见缝插绿，拆旧造绿，扩大中心区公共绿地面积，抓好南寨公园二期、玉门河公园、学府公园、植物园建设。在南部新区和主要出口路两侧高密度、大规模植树造林，开展城市周边生态林建设，继续实施退耕还林、天然林保护、三北防护林工程，抓好东西山绿化、交通沿线绿化、荒山荒坡绿化、农村村镇绿化和采煤沉陷区植被恢复绿化。完成造林100万亩，培育中幼林50万亩。建立生态补偿机制，实行企业环境治理和生态恢复责任制。今年重点抓好环城林带、机场大道、西北环连接线、滨河东西路、长风东大街和大东关连接线绿化，提高档次，形成景观。通过几年的不懈努力，使太原的天更蓝，水更清，气更新，草更绿，花更艳，努力建成全国生态园林城市，实现人与自然的和谐相处和良性互动。

七、加快体制机制创新，提高对外开放水平

在经济社会的各个领域深化改革，努力突破、消除体制性障碍；以开放促改革、促发展，不断增强经济社会发展的内在动力和活力。

推进国有企业实质性改革，使国有经济整体上焕发生机。按照“让开两厢、重点突破、滚动改制”的思路，通过破产拍卖、产权转让、收购兼并、内引外联等多种形式，大力推进市属国有企业改革。采取财政贴息，争取银行贷款，建立专项资金等措施支持国企改革，企业土地出让收益和资产变现进入专户管理，全部用于企业改革，解决好职工补偿、股权转让、社保接续、主辅分离、辅业改制和企业办社会等问题，为企业改革扫清障碍，用2年时间打赢国企改革攻坚战。建立健全国有资产监督管理体制和监管方式，完善出资人制度，实行国有资本经营预算和企业经营业绩考核制度，确保国有资产保值增值。加强企业家队伍建设，培育一批独领风骚的现代“晋商”。

放手发展非公有制经济，培育具有竞争活力的增长点。清除政策性、体制性障碍，放宽企业经营范围，降低市场准入门槛，鼓励民营资本进入社会公用事业、基础设施建设等领域，支持民营企业参与国有、集体企业的改组、改制、改造。引导非公有制企业建立现代企业制度，提升产业层次和企业整体素质。“十一五”期间，非公有制经济年均增长18%，到2010年占GDP的比重达到50%以上。

加快投融资体制改革，构建筹资融资的有效机制。加大对农业、中小企业的信贷支持力度，发展为中小企业、民营企业提供金融信贷服务的担保机构。推进投资体制改革，不使用政府投资的建设项目，政府一律不再审批，区别情况实行核准制和备案制，落实企业投资自主权。树立经营城市理念，全面放开城市基础设施建设市场，有偿转让国有资产和经营权，盘活城建经营性资产。要尽快搭建融资平台，抓好城建资本运营，加大项目融资力度，多渠道筹集建设资金。搞好土地市场化运作，不断提高国土收益。改革政府投资工程管理方式，按照“投资、建设、管理、使用”分离的原则，推行代建制，降低工程造价，提高投资效益。加快建设系统企事业单位改革，企业单位脱钩转制，事业单位企业化改制。加快市政公用行业改革，全面放开作业市场，努力实现市政、园林、环卫、公交、供热、自来水、污水处理等企事业单位的市场化运营。

深化财政管理体制改革，发挥公共财政的最大效能。以建立公共财政体制为目标，改革财政管理体制，科学划分收支，壮大县级财力。调整优化支出结构，优先保证并向社会事业和“三农”倾斜，财政新增教育、卫生、文化等事业经费主要用于农村。深化部门预算改革，完善预算编制内容，提高预算编制的科学性。加大国库集中支付制度改革力度，取消各类收入过渡性账户，建立以国库单一账户体系为基础的财政国库管理制度，市级单位全部纳入国库集中支付范围。扩大政府采购范围和规模，完成政府采购采管分离改革。

推进事业单位改革，探索与社会经济发展相适应的新体制。按照分类指导、稳步推进的原则，加快事业单位人事制度、分配制度、产权制度、社会保障制度改革，建立政事职责分开、单位自主用人、人员自主择业、政府依法管理、配套措施完善的分类管理体制。全面建立和推行聘任制度，促进事业单位人事管理由固定用人向合同用人转变，由身份管理向岗位管理转变，由行政管理向法制管理转变。抓好高层次和紧缺人才的培养、引进和使用，提高各类人才的学习、实践和创新能力。加强人才服务工作，建立门类齐全、素质优良、梯次合理、新老衔接的人才队伍，营造人才辈出、人尽其才的社会氛围。

敞开胸怀招商引资，让太原成为创业者的乐园。进一步创优招商引资环境，减少和规范行政许可，压缩审批时限，提高办事效率。完善并严格执行审批事项公开制、首办负责制、审批服务承诺制、行政不作为或乱作为追究制等一系列制度，切实提高行政效率和服务水平。对外商投资企业所有审批手续实行限时办结，坚决整治各种乱收费、乱罚款、乱摊派和刁难投资者等行为。完善招商引资项目库，拿出好的项目和优质资产招商引资。积极参加各种大型经贸洽谈活动，开展园区招商、网上招商、委托招商、以商引商。成立招商引资工作领导组，统一组织、指导全市重大招商引资活动，协调解决对外开放和利用外资中的重要问题。制定招商引资奖励办法，把招商引资列入目标责任制考核范围，实施重大项目领导联系分包制和专人负责制，狠抓签约项目的落实，提高合同履约率、资金到位率和项目成功率。“十一五”期间，力争引进世界500强15家以上，引进国内200强企业20家以上。

加强对外贸易和经济技术交流合作，努力提高经济外向度。依托资源、劳动力优势，扩大对外工程承包和劳务服务，把加工贸易培育成出口的主要增长点。鼓励企业进行质量、环境和产品认证，提高进入国际市场的能力。支持民营企业扩大出口贸易，进一步形成国有、外资、民营为主体的出口格局。加强对外贸企业的监督管理，规范进出口经营行为。建立出口产品扶持制度，优化出口产品结构，扩大装备制造、新材料、电子信息等技术含量高、附加值高、加工程度高的产品出口比重。按照省委、省政府的要求，积极构建“太原经济圈”。加强与京津冀、环渤海经济圈和中部地区的联系与合作，不断扩大产业发展空间和市场空间。

加强市场体系建设，促进生产要素公平自由流动。健全以市域商业中心为主导、区域商业中心为主体、社区村镇商业网络为基础、商业街为特色，布局合理，业态优化，功能齐全的商业体系。大力发展资本、土地、技术、信息、劳动力等要素市场，充分发挥市场对资源配置的基础性作用。积极发展独立公正、规范运作的市场中介服务机构，发挥各类行业协会和商会组织的作用。以质量监督、药品监督、价格监管和食品卫生安全为重点，大力整顿和规范市场经济秩序。推进社会信用体系建设，建立健全鼓励守信、惩戒失信的信用监督约束机制，完善统一的企业信用信息数据征集、评价和发布平台，营造良好的信用环境，努力打造“信用太原”。

八、推进和谐太原建设，全面发展社会事业

把群众利益作为各项工作的根本出发点和最终归宿，突出解决好人民群众最关心的就业、社会保障、教育、医疗和安全等问题，创造公平公正、安全稳定的社会环境，构建以人为本的和谐社会。

广辟就业渠道，努力让能做事者工作有保障。把扩大就业作为政府宏观调控的重要目标，实施积极的就业政策。多渠道开发就业岗位，加大开发购买、补贴购买公益岗位的力度，大力开发社区岗位。积极稳妥地做好出中心人员的身份置换、社保接续和就业安置工作，今年完成下岗职工出中心并轨任务。认真落实再就业扶持政策，发挥再就业服务平台作用，支持下岗失业人员自主创业，实现多种形式就业。“十一五”期间，年均新增城镇就业岗位14万个。

完善社保体系，努力让应救助者生活有保障。建立与经济发展水平相适应的制度完善、管理科学、体系健全的社会保险制度和管理服务网络。增加财政的社会保障投入，多渠道筹措社会保障基金，做实个人账户。加大基金征缴清欠力度，扩大社会保险覆盖面，逐步将城镇各类劳动者纳入基本社会保险统筹范围。 “十一五”期末，企业基本养老保险、机关事业单位养老保险覆盖率分别达到97%和100%，医疗保险覆盖率达到90%，生育保险覆盖率达到76%，工伤保险覆盖率和煤矿职工意外伤害保险覆盖率分别达到76%和100%，失业保险覆盖率达到99%，退休人员社会化管理服务率达到99%，住房公积金制度覆盖率达到95%。提高企业离退休人员、城市“低保”对象、军队移交政府安置的离退休人员、残疾军人等各类优抚对象的生活补助标准。全方位构筑社会保障网，进一步完善以城乡最低生活保障、医疗救助、教育救助、灾害救助、法律援助、廉租住房、农村五保供养、特困户救济为主要内容、覆盖城乡的社会救助体系，维护妇女儿童权益，发展残疾人事业，实现幼有所护、老有所养、弱有所助、残有所扶、难有所帮，让党和政府关怀民生的阳光洒遍社会的每一个角落，温暖每个困难家庭和群众的心房！

优化教育结构，努力让适龄青少年上学有保障。按照“三个面向”的要求，积极构建现代国民教育体系。义务教育要在加快企业学校剥离的基础上，大力整合教育资源，加快学校布局调整，重点扶持薄弱学校，新建和改造100所优质示范性中小学，促进义务教育均衡发展，切实解决好孩子上学难、择校热的问题。对城乡特困学生继续实行“两免一补”，逐步在全市实现免费义务教育，小学适龄儿童入学率、初中毛入学率达到100%，城区义务教育标准化学校达到100%。重视发展城乡幼儿教育，巩固学前三年教育普及成果。高中阶段教育要按照普职融通原则，继续抓好普通高中标准化、示范性建设，大力发展职高、中专、技校为载体的中等职业教育，全市新建和改造一批重点职业学校，建设实习和实训基地，每个县区至少办好一所多功能职业教育中心，形成以就业为导向，以服务为宗旨，校企合作、产教结合、工学结合的职业教育发展新机制。高等教育要通过整合市属高校教育资源，形成以高职高专为主的2+X模式的高等教育体系。抓好教育园区建设，把太原大学建成体制灵活、特色鲜明，多学科、开放式的综合大学。

实施“康民”工程，努力让全民健康水平有保障。医疗卫生要以构建公共卫生体系为主，整合卫生资源，形成政府主导、民办为辅、以城带乡、城乡一体的医疗卫生服务新格局。加大政府投入，建设惠民医院，坚决制止医疗乱收费、高收费，彻底整治行业不正之风，使群众不再为看病难、看病贵犯愁。深化医疗卫生体制改革，健全和完善医疗服务、疾病预防控制、急救网络和卫生监督体系，增强突发公共卫生事件的反应、处理和救治能力。预防控制重大传染病和高致病性禽流感等动物疾病，保护人民群众的身体健康和生命安全。加强妇幼卫生保健，降低出生缺陷发生率。完善社区卫生服务体系，社区卫生覆盖率达到95%。人口与计划生育工作要拓展服务范围，稳定低生育水平，改善人口结构，提高出生人口素质。广泛开展全民健身运动，加快群众体育活动场所和设施建设，改造一批体育场馆，全市所有社区和60%以上的乡镇建立体育健身点和指导站，体育人口达到50%以上。提高竞技体育水平，积极申办国际国内体育赛事，举全市之力，精心组织好2008年小轮车世锦赛暨奥运会预赛。

严格安全责任，努力让群众生命财产有保障。坚持安全第一、预防为主、综合治理的方针，加大安全投入，夯实安全基础，健全安全生产监管体制，严格落实安全生产责任制，突出抓好煤矿、非煤矿山、危险化学品等行业的安全整治和道路交通安全、消防安全。加强对重大危险源的排查、监控和整改，实行事故隐患整改责任追究制度，加大各类事故的责任追究和处罚力度，有效遏制重特大事故发生，促进安全状况明显好转。

加强国防后备力量建设，增进军政军民团结，继续创建“双拥模范城”。认真组织实施农业普查，高质量完成各项统计任务。“十一五”期间，人防、气象、地震、档案、地方志等各项工作和老龄、妇女、关心下一代等各项事业，都要实现新发展，取得新成绩。

九、加强社会主义民主政治和精神文明建设，为经济社会发展提供政治保障和精神动力

在推进经济社会快速健康发展的同时，大力发展社会主义民主政治和先进文化，促进物质文明、政治文明和精神文明全面进步，为构建民主法治、文明和谐社会奠定坚实基础。

立足推进依法治市进程，努力建设民主法治社会。各级政府要定期向人大及其常委会报告工作，自觉接受人大常委会和人民代表的法律监督和工作监督，主动接受政协的民主监督、媒体的舆论监督、群众的社会监督，认真听取各民主党派、工商联、无党派人士和人民团体的意见。积极办理人大代表建议和政协提案。支持人民法院、检察院依法履行职责。充分发挥工会、共青团、妇联等人民团体的桥梁纽带作用。重视做好民族、宗教和侨务工作。加强基层民主建设，完善政务、厂务、村务公开和基层民主选举制度，保证基层群众依法行使选举权、知情权、参与权和监督权。深入开展“五五”普法教育，提高全民法律素质。认真贯彻《信访条例》，实行市、县、乡三级领导信访接待日制度，落实信访责任，畅通信访渠道，引导群众以理性合法的形式表达诉求，切实解决群众反映强烈的热点难点问题。做好社会矛盾纠纷排查调处工作，积极预防和妥善处置群体性事件。加强社会治安综合治理，引深平安创建活动，依法严厉打击各类违法犯罪活动，增强人民群众的安全感。健全社会预警体系和应急机制，加强自然灾害监测和预警，加快应急基础平台建设，搞好应急联动演习，提高应对突发事件能力和防灾减灾能力。

面向大众丰富精神文化产品，在全社会力倡文明新风。繁荣哲学社会科学研究、新闻出版、广播电视、文化艺术，为人民群众提供更多更好的精神文化产品。充分发挥市群众艺术馆、县文化馆、乡镇（街道）文化站、社区（村）文化点四级文化网络作用，逐步形成完备的公共文化服务体系。加强文物、文化遗产、民间艺术保护和文化市场管理，营造扶持健康文化、抵制腐朽文化的社会环境。强化理想信念教育，使“三个代表”重要思想和科学发展观深入人心，弘扬以爱国主义为核心的民族精神和以改革创新为核心的时代精神，树立以“八荣八耻”为主要内容的社会主义荣辱观，建立完善社会公德、职业道德、家庭美德和个人品德等思想道德体系，在全社会形成爱国守法、明礼诚信、团结友善、勤俭自强、敬业奉献的基本道德规范。发扬“兼容和谐、诚信卓越”的城市精神，深入开展精神文明创建活动，不断提高城乡文明程度，努力把太原建成全国文明城市。

十、深化行政管理体制改革，切实加强政府自身建设

加快转变政府职能，建立行为规范、运转协调、公正透明、廉洁高效的行政管理体制，认真贯彻《公务员法》，创新管理方式，提高工作效率，为圆满完成“十一五”的各项目标任务提供有力保障。

变观念，树立敢为人先新意识。以邓小平理论、“三个代表”重要思想和科学发展观为准绳，以是否促进经济社会发展和人民群众生活水平提高为检验标准，坚决破除一切不合时宜、不利于发展的思想观念，坚决改变一切束缚发展的做法规定，坚决扫除一切影响发展的体制障碍。破除小胜即满、小富即安的自满意识，树立永不满足、勇创一流的进取精神；破除墨守陈规、保守僵化的思想束缚，树立实事求是、敢闯敢干的创新精神；破除按部就班、不紧不慢的平庸思想，树立不甘落后、敢为人先的开拓精神；破除怕苦畏难、无所作为的懒散作风，树立知难而进、百折不挠的拼搏精神；破除本位主义、各自为政的狭隘观念，树立顾全大局、同心协力的团队精神，在全市形成改革创新、干事创业、奋发有为的良好氛围。

转职能，形成履行职责新行为。各级政府要切实履行经济调节、市场监管、社会管理、公共服务的主要职能，推进政企分开、政资分开、政事分开、政府与市场中介组织分开，坚决把政府不该管、也管不好的事交给市场、企业、社会组织和中介机构，努力建设服务型政府；坚持民主科学决策，完善公众参与、专家论证和政府决策相结合的决策机制，明确决策机关的权限和程序，实行重大问题集体决策制度、专家咨询制度、社会公示、社会听证和新闻发布制度，努力建设“透明”政府；优化政府组织结构，规范市县两级政府和政府组成部门的事权和职责，提高政府执行力，努力建设高效政府；坚持依法行政，按照法定权限和程序行使权力、履行职责，健全行政责任制、绩效考核制和对行政管理权限的监督机制，以严格的管理，严明的纪律，保证政府的公信力，努力建设法治政府；着眼于新的实践和发展，坚持与时俱进，倡导创新精神，创造性地开展工作，努力建设创新型政府；加强廉政建设，强化审计监督，建立健全教育、制度、监督并重的惩治和预防腐败体系，从源头上预防和治理腐败。坚决治理工程建设、土地出让、产权交易、医药购销、政府采购、资源开发和经销领域的商业贿赂行为，努力建设廉洁政府。

倾真情，塑造亲民爱民新形象。各级政府干部和公务员要牢记全心全意为人民服务的宗旨，自觉实践“以民为根本、敬民如父母”的从政理念，切实做到权为民用，情为民系，利为民谋，始终对群众充满感情，把实现群众愿望、落实群众需要、维护群众利益作为一切工作的出发点和落脚点，坚持群众的利益高于一切，群众的冷暖急于一切，群众的呼声先于一切，多为群众办实事、好事，解决实际问题。在开展工作中更加注重社会效果，在推进改革时更加注重保障群众利益，在经济发展中更加注重利民富民，让人民群众每年都能分享到增加收入、提高生活质量的喜悦，为全市340万人民的福祉，殚精竭虑，鞠躬尽瘁，用真心、真情和实干赢得人民群众的信赖和支持！

讲效能，励炼立说立行新作风。以贯彻实施《公务员法》为契机，建设一支政治坚定、业务精湛、作风过硬、人民满意的公务员队伍。各级政府干部和全体公务员要不断提高贯彻科学发展观的能力、驾驭全局的能力、处理利益关系的能力和务实创新的能力。深入开展效能建设，坚持做到立说立行，雷厉风行，加快节奏，提高效率，坚决改变推诿扯皮、办事拖拉的不良作风；身体力行求真务实之风，坚持重实际、讲实话、出实招、求实效，力戒心浮气躁，力戒坐而论道，力戒急功近利，不搞形象工程，不做表面文章，切实做到为人民谋福利，为人民创政绩；着力提高工作水平，坚持高标准，追求高质量，发扬“逢一必争、逢冠必夺”的奥林匹克精神，各项工作都要千方百计争第一、当排头、创一流，走在全省乃至全国前列。要紧紧围绕改革发展稳定中的突出问题、经济运行中的突出问题、关系群众切身利益的突出问题，勤于思考，精于预见，善于应变，增强工作的超前性和主动性，抓住关键环节，突破重点难点，以点带面，推动全局。要形成一级抓一级、一级对一级负责，环环相扣、层次分明的责任体系，加大对重点工作、重点项目和重点目标的督促检查力度，促进各项工作顺利进行。

各位代表，今年是“十一五”规划的开局之年，我们要按照本次大会批准的“十一五”规划纲要，以只争朝夕的精神，抓紧推进建设创新型城市、实现率先发展的各项工作，经济社会发展指标都要好于规划提出的目标任务，为“十一五”规划的全面实施开好局、起好步、奠定坚实基础。在扎实做好各项工作、全面完成省政府规定的十二件实事的同时，今年市政府要着力解决人民群众反映强烈的热点、难点问题：一是着力解决发展环境中的突出问题，深入开展“环境创新年”活动，提高行政效能和服务质量，营造良好的投资发展环境；广泛开展环境卫生整治活动，营造良好的市容环境；实施畅通工程，营造良好的出行环境；加大环保力度，市区二级以上天数达到260天。二是着力解决上学难的问题，建设农村高标准九年一贯制学校和寄宿制中心小学15所，免除全市农村义务教育阶段学生学杂费；新建、扩建和改造城市优质示范中小学20所，完成100所城镇中小学活动场地改扩建工作，实现优质教育均衡化和普及化。三是着力解决看病难的问题，全面实行农村新型合作医疗制度，参合率达到80%以上；为54个乡镇卫生院配置X光机、B超等设备；由政府出资把一批社区卫生服务机构纳入医保，租赁房屋的每家补助2万元资金；完善困难群众医疗救助制度；设立一所惠民济困医院，实行收支两条线管理，为困难群众提供医疗服务。四是着力解决住房难的问题，建设安康居住工程90.5万平方米，解决8493户困难居民的住房；改造工矿企业棚户区，开工建设住宅面积21万平方米，解决3348户棚户住房；治理矿山沉陷区，新建住宅面积43万平方米，解决7158户居民住房；维修加固城乡住房44.6万平方米，让7863户城镇居民和1000户农村特困群众住进安全的房子。五是着力解决行路难的问题，高标准、高质量完成33条城市主要道路的续建、新建和大修任务，对53条小街小巷实施改造，所有小街小巷安装路灯，提高道路质量和亮化美化水平；实施村村通水泥（油）路和村村通客车工程，修建水泥（油）路270公里，97%以上的建制村通油路、通客车。六是着力解决就业和社会保障难的问题，新增城镇就业岗位11万个，下岗失业人员再就业4.4万人，安置“4050”人员6733人；培训农民5万人，转移农村富余劳动力4万人；农村低保覆盖率提高到5%。新建和改造敬老院，用两年时间，使农村五保户全部实现集中供养。

各位代表，太原已跨入充满希望的“十一五”时期，龙城腾飞的壮丽前景召唤我们乘胜奋进，晋阳人民的美好期盼激励我们昂首前行。让我们紧密团结在以胡锦涛同志为总书记的党中央周围，在省委、省政府和市委的坚强领导下，全面贯彻落实“三个代表”重要思想和科学发展观，群策群力，团结拼搏，咬定目标，扎实推进，为实现国民经济和社会发展第十一个五年规划和全面建设小康社会的宏伟目标而努力奋斗！
